# Is That Me or My Twin? Lack of Self-Face Recognition Advantage in Identical Twins

**DOI:** 10.1371/journal.pone.0120900

**Published:** 2015-04-08

**Authors:** Matteo Martini, Ilaria Bufalari, Maria Antonietta Stazi, Salvatore Maria Aglioti

**Affiliations:** 1 Department of Psychology, University of Rome “La Sapienza,” Via dei Marsi 78, 00185 Rome, Italy; 2 IRCCS Fondazione Santa Lucia, Via Ardeatina 306, 00100 Rome, Italy; 3 Institut d'Investigacions Biomèdiques August Pi i Sunyer (IDIBAPS), Carrer del Rosselló, 149, 08036 Barcelona, Spain; 4 School of Psychology, University of East London, Water Lane, Stratford, London E15 4LZ, United Kingdom; 5 National Twin Registry—Italian National Institute of Health, Viale Regina Elena 299, 00161 Rome, Italy; Bournemouth University, UNITED KINGDOM

## Abstract

Despite the increasing interest in twin studies and the stunning amount of research on face recognition, the ability of adult identical twins to discriminate their own faces from those of their co-twins has been scarcely investigated. One’s own face is the most distinctive feature of the bodily self, and people typically show a clear advantage in recognizing their own face even more than other very familiar identities. Given the very high level of resemblance of their faces, monozygotic twins represent a unique model for exploring self-face processing. Herein we examined the ability of monozygotic twins to distinguish their own face from the face of their co-twin and of a highly familiar individual. Results show that twins equally recognize their own face and their twin’s face. This lack of self-face advantage was negatively predicted by how much they felt physically similar to their co-twin and by their anxious or avoidant attachment style. We speculate that in monozygotic twins, the visual representation of the self-face overlaps with that of the co-twin. Thus, to distinguish the self from the co-twin, monozygotic twins have to rely much more than control participants on the multisensory integration processes upon which the sense of bodily self is based. Moreover, in keeping with the notion that attachment style influences perception of self and significant others, we propose that the observed self/co-twin confusion may depend upon insecure attachment.

## Introduction

Self-face recognition is crucial for sense of identity and for building and maintaining self-awareness [1–4]. Moreover, self-face recognition has to do with introspection, which may be a cognitive pre-requisite of our ability to infer the mental states of others [5,6]. Converging behavioral, neuropsychological and cognitive neuroscience studies have shown that one's own face has a robust representation ([7], but see also [8]) and receives special brain processing, at least in the species that are endowed with self-recognition abilities.

One’s own face grabs [9] and retains [10] attention longer than other identities. Importantly, people respond to and recognize faster one’s own face with respect to famous [11,12] and friend’s faces [13–16]. This self-face advantage is found for both upright and inverted faces [13]. Upright faces are typically processed holistically (or configurally, i.e. as a whole, a ‘gestalt’), while inverted faces are processed locally (i.e. through a “part-based recognition”, see [17,18]). Usually, people are generally better at recognizing images of upright compared to inverted faces (i.e., the face inversion effect [19]), but local features are more frequently used for the judgment of one's own face. Thus, not only configural information, but also featural information can make important contributions to self-face processing [20].

At a neural level, processing of the self-face is distinguishable from processing of other faces, even highly familiar ones [21]. Self-face processing seems to rely upon a right-dominant but largely bilaterally distributed circuit involving occipito-temporo-parietal and frontal regions [22–26]. Also neuropsychological evidence points toward a robust representation of one’s own face. Indeed, despite the loss of the capability to recognize very familiar faces (as in prosopagnosia) genuine deficits in one’s own face recognition have been reported very rarely and almost exclusively in patients with severe neurological [27,28] or psychiatric disorders [29–31]. Therefore, as it is crucial for recognizing the bodily self and inherently linked to identity, one's own face is robustly represented in the human cognitive and emotional system.

Monozygotic twins’ faces represent a rare exception to the uniqueness of the self-face: a monozygotic twin’s face shares almost all physical features with another non-self face that is highly emotionally relevant and personally known since birth. Thus, monozygotic twins are a unique population to explore self-face recognition and self-identity processes because of their extreme physical similarity and familiarity with one another’s face.

It has been suggested, for example, that monozygotic twins may encounter difficulties in the self-identification process, as they have a stronger sense of identity as a couple than as an individual [32]. Preliminary studies reported that identical twins might sometimes mistake their own face for the face of their twin [33]. While the ability to recognize oneself in a mirror is already generally developed at approximately age two [34], studies indicate that two-years old twins discriminate their own faces from the face of their co-twins only if exposed to the facial stimuli for a long period of time [35]. Recently, twins’ faces have been used as experimental visual stimuli [36–39], and twins have been employed as experimental subjects [39–42]. However, thus far no empirical evidence has been reported about the ability of adult twins to discriminate their own face from the face of his/her co-twin.

In light of these premises, the main objective of the present study was to examine at both configural and featural processing levels whether despite their physical similarity, twins would be better at recognizing their own face compared to their co-twins’ face. To address this issue we devised a task in which monozygotic twins were asked to report by pressing one of three keys whether upright and inverted facial pictures were of themselves or the co-twin. As a control, we also included a non-twin face to test whether twins were better at recognizing their co-twins’ face or another personally well-known face, which was matched as much as possible for familiarity, exposure, and emotional valence, all factors that play role in face recognition processes [43–46]. For each pair of twins, a non-twin individual was included in a control group and tested with the self and the two twins’ faces. Using upright and inverted faces allowed us to explore the influence of configural and featural processing levels in modulating the self-face advantage, or lack of it.

Studies indicate that face recognition can also be influenced by different personality characteristics. For instance, self-face processing in the right hemisphere is impaired in individuals with schizotypal traits [47]. On the other hand, extroverts who typically have better social skills recognize a higher number of faces than introverts [48]. Moreover, face recognition is negatively correlated with social anxiety, suggesting that lower face recognition is associated with increased social phobia [49]. Thus, we also explored whether the personality of the twins might play a role in the recognition of their own faces, as it has been previously been suggested for non-twins [50]. To this aim we correlated personality measures with performance scores in the experimental tasks. In particular, given the special emotional link between identical twins [51,52], we hypothesized that their attachment style might influence their performance in discriminating the self face from the twin’s face. Studies indicate that exposure, familiarity and physical similarity play a role in recognizing one's own and others' face [53,54]. Thus, for each participant we measured the length of acquaintance, how frequently they saw each other and the subjective ratings of physical resemblance of one’s own face with the other two faces.

## Method

### Participants

Thirty participants were recruited for this study: 10 monozygotic, right-handed, gender-balanced, healthy twin couples (mean age ± SD: 21.4 ± 1.8 years, range 18–23) and 10 right-handed, gender-balanced, healthy controls (mean age ± SD: 22.5 ±3.8 years, range 20–32). All subjects belonged to the same race (white-Caucasian). Each control participant was a sex-matched, close friend (n = 8) or relative (n = 2: 1 cousin and 1 brother) of a given twin pair. Each control participant had known the twins in the relative pair for at least 3 years (mean relationship duration = 14 years) and had daily interaction with them (on average 9 controls regularly met the twins 7 days per week, while 1 control met them 5 days per week).

The twins were recruited from the “National Twins’ Register” of “Istituto Superiore di Sanità (I.S.S.)” of Rome. Zygosity was previously determined by the I.S.S. through a standard interview process that has proved to be highly reliable [55].

Before starting the experiment, participants signed an informed consent and received a monetary compensation at the end of the task. The procedures were approved by the local ethics committee of Fondazione Santa Lucia and were in accordance with the ethical standards of the Declaration of Helsinki. The individuals in this manuscript have given written informed consent (as outlined in PLOS consent form) to publish these case details.

### Stimuli and apparatus

Subjects were tested in two days. On the first day, pictures of the subjects’ faces were taken. To eliminate the influence of local cues, female subjects were asked not to wear make-up and male subjects were asked to be clean-shaven. A Canon 450-D camera (Canon Inc.© Ōta, Tokyo, Japan) was used to take the pictures. Subjects were instructed to display a neutral facial expression. Pictures were cropped into an oval frame to eliminate non-facial cues. Pictures were then converted to grayscale and centered on a black background.

**Fig 1:**
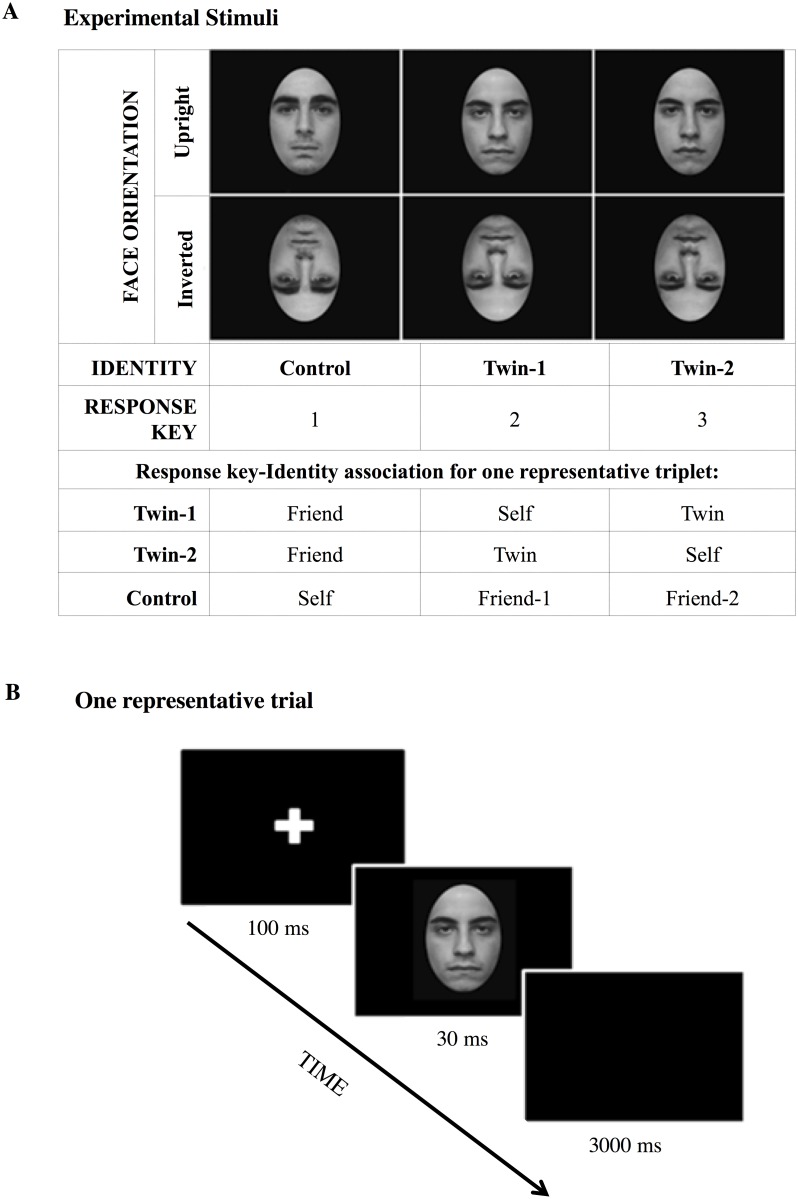
Experimental stimuli and procedure. A) Example of the experimental conditions and stimuli for each trio (twin 1, twin 2, and control participant). Each twin was presented with the Self, the Twin and the Friend Face, while the control participant was presented with the Self and both the twins’ faces. Each key (1, 2, 3) was associated with one of the three identities (Control, Twin 1, Twin 2), regardless of the orientation (upright, inverted). Thus, in the represented trio, Twin-1 responded with key 1 for the Friend face, with key 2 for the Self-face and with key 3 for the Co-twin face. Twin-2 responded with key 1 for the Friend face, with key 2 for the Co-twin face and with key 3 for the Self-face. Control participant responded with key 1 for the Self-face and with keys 2 and 3 for the Twin-1 and Twin-2 faces, respectively. B) Schematic representation of one representative trial. A black screen with a white cross (1 sec.) preceded the display of one of the three faces (30 ms.), followed by a black screen (3 sec.), during which the subject had to respond which identity he/she had seen by pressing the associated response key. The individuals in this manuscript have given written informed consent (as outlined in PLOS consent form) to publish these case details.

Upright and inverted (180°) faces were created for each identity. Thus, each participant was presented with three identities (TWIN group: Self, Twin, Friend; CONTROL group: Self, Friend-1 (i.e. one of the twins), Friend-2 (i.e. the other twin)) and two orientations (upright; inverted; see Fig. 1A). Self-face pictures were presented as mirror images; that is, in the way subjects were used to seeing themselves in mirrors (flipped left-right). Digital manipulations were performed with Photoshop 6.0 (Adobe Systems Incorporated, San Jose, CA, USA). On the second day, subjects performed the recognition task and completed the various questionnaires.

### Design and procedure

Subjects sat approximately 60 cm from a 17-inch computer screen. All subjects had normal or corrected-to-normal vision. Visual stimuli were presented randomly by means of E-Prime 2.0 (Psychology Software Tools Inc., Sharpsburg, USA). Each trial started with the presentation of a fixation cross (1 sec.) in the center of a black screen, followed by a rapid (30 ms) appearance of one of the six stimuli. A pilot study revealed that for stimuli presentations of 60 ms, upright pictures of either self, the co-twin’s and the friend’s faces were subjected to ceiling effects (all “hits” between 90 and 100% of accuracy). The 6 subjects participating in the pilots were not taken in consideration for the final experiment.

The six stimuli comprised one inverted and one upright image of the face of each of the three participants in the trio (Fig. 1A). After the facial stimulus a black screen appeared (up to 3 sec.; Fig. 1B); during this time the subject reported the identity of the face by pressing the “1”, “2” or “3” key with their right index-, middle- or ring-finger, respectively.

Within each trio of subjects (the two twins and the control participant), each number (finger) was assigned to represent one of the three participants. So, for instance, for all the participants in the trio the key “1” always indicated the twin “Mario”, the key “2” the twin “Marco” and the key “3” their friend “Giovanni”. This number-identity association was counter-balanced across each group.

Participants were asked to identify each face displayed on the screen as quickly and as accurately as possible. There were six different conditions in total: the three identities in each trio (Twin-1, Twin-2, Friend) displayed either upright or inverted. Overall, a total of 336 pictures (56 images per condition) were displayed in 4 sessions (84 stimuli in each session, 14 stimuli of the same condition in each session), separated by three resting pauses. A short training session (12 pictures, no time constraints) preceded the real experiment. To rule out possible effects on performance caused by forgetfulness, a written reminder with the correct key–name association was visible to the subjects throughout the whole task.

### Personality tests and subjective reports about the experimental stimuli

Before the experiment, each participant was administered the Italian version [56] of the Attachment Style Questionnaire (ASQ; [57]) reflecting the respondents’ adult attachment styles. The ASQ is a 40-item, Likert-type, self-administered questionnaire designed to measure five dimensions of adult attachment: “Confidence” (8 items), “Discomfort with Closeness” (10 items), “Need for Approval” (7 items), “Preoccupation with Relationships” (8 items) and “Relationships as Secondary” (7 items). Each item was rated on a 6-point scale, ranging from 1 (totally disagree) to 6 (totally agree). To avoid response bias, the items were listed in random order and three items were reverse-scored. The ASQ showed adequate reliability and construct validity in university and secondary student samples [57].

Subjective measures on the perceived physical similarity (resemblance) between the rater’s face and each of the other two faces were provided along a Visual Analogue Scale (VAS) ranging from 0–10, with “0 = not resembling my face at all” and “10 = fully resembling my face”. Participants were asked to place a mark on the continuum to indicate their judgment of physical similarity for each face. Familiarity judgments with the other two identities (e.g., “length of acquaintance” measured in months (coincident with age); and “frequentation” (days per week spent together)) were also collected.

### Data handling

Accuracy and reaction times for upright and inverted pictures were collected. Incorrect responses were discarded from the data analysis. To obtain an integrated measure of performance, response times were divided by the proportion of accurate responses per condition and subject. In this way, inverse efficiency scores (IE) were obtained [58], allowing us to control for any speed-accuracy trade-off effect.

For all variables, the assumption of normality was assessed by means of the Kolmogorov-Smirnov test. Some IE scores did not pass the test (p<0.05). Therefore, all IEs were normalized according to a 1/X ratio, where “X” represents the subject’s IE score, obtained for each variable. This procedure is known to be particularly effective in adjusting positively skewed distributions [59]. It is worth noting that the lower the value of the normalized score, the worse the performance.

To assess if the self-face had an advantage in the Control and the Twin groups, two repeated measures ANOVAs on the normalized inverse efficiency scores (one for the control and one for the twin group) were run. The factors of the ANOVAs were the following: Identity (respectively: “Self”, “Friend-1”, “Friend-2” for the Control group; and “Self”, “Co-Twin”, “Friend” for the Twin group) and Orientation (“Upright”, “Inverted”). Newman-Keuls post-hoc comparisons were used when significant main or interaction effects were found.

In two male subjects, the scores in the “Upright” face, “Co-Twin” condition were clearly deviant from the group mean (beyond 2.5 SD from the group’s mean) and thus were replaced with the group’s mean value [59].

The potential link between personality, “Interpersonal Perception” (level of acquaintance, frequentation, resemblance) and the twins’ self-face discrimination ability was assessed through two separated standard regression models, one for the personality measures and the other for acquaintance, frequentation and resemblance.

## Results

### Twins lack self-face recognition advantage

**Fig 2:**
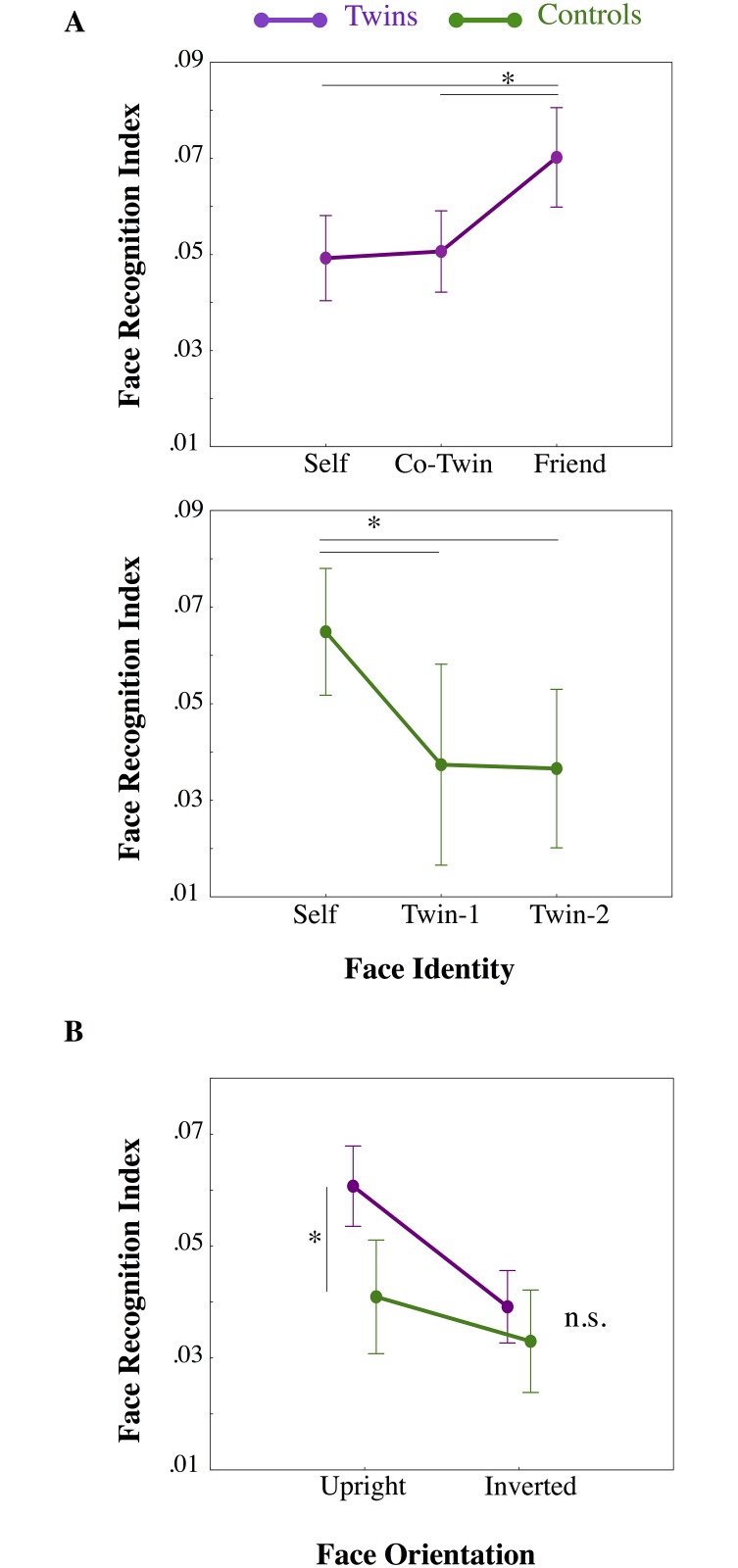
Face recognition indices. A) Results of separated repeated measures ANOVAs on Face Recognition performance (normalized inverse efficiency scores (IE), y-axis) scores are represented for both Twins (violet line) and Control (green line) participants as a function of observed Face Identity (x-axis). B) Results of mixed-model ANOVA comparing Twins (violet line) and Control (green line) Face Recognition performance (normalized inverse efficiency scores (IE) for mediated Twins faces; y-axis) as a function of Face Orientation (x-axis). Means and standard errors (SE) are represented. Asterisks indicate significant differences from Newman-Keuls post-hoc comparisons.

Results of the repeated measure ANOVA in the Twin group showed a main effect of Orientation (F_1,19_ = 95.441, p = 0.000; η^2^ = 0.834) with better performance in recognizing Upright (mean ± SD: 0.068 ± 0.013) with respect to Inverted face stimuli (0.047 ± 0.021; p = 0.000). Also, a main effect of Identity was found (F_2,38_ = 22.929, p = 0.000; η^2^ = 0.547) with similar performance in recognizing the self (0.052 ± 0.018) and the co-twin’s (0.051 ± 0.016) faces, which were both different from the friend face (0.07±0.018) (both comparisons, p = 0.000) (Fig. 2A, upper panel). No significant interaction between the two factors was found (F_2,38 =_ 0.296; p = 0.746).

Results of the repeated measure ANOVA in the Control group also showed a main effect of Orientation (F_1,9_ = 59.412, p = 0.000; η^2^ = 0.868), with better performance in recognizing Upright (0.052 ± 0.023) with respect to Inverted (0.041 ± 0.022; p = 0.000) face stimuli and a main effect of Identity (F_2,18_ = 25.183, p = 0.000; η^2^ = 0.737) showing that, unlike twins, controls were better at recognizing their own face (0.065± 0.017) than both Friend-1 (0.037 ± 0.021; p = 0.000) and Friend-2 (0.037 ± 0.019 p = 0.000) faces (in turn not different from one another, p = 0.861) (Fig. 2A, lower panel). No significant interaction between the two factors was found (F_2,18 =_ 0.606; p = 0.556).

Since the twins’ faces were highly resembling and difficult to discriminate from each other (both groups performed similarly with the two twin faces), we re-analyzed the data by comparing the performance of the two groups with the average of the two twins’ faces using a mixed model ANOVA with Group (Twins; Controls) and Orientation (Upright; Inverted) as between and within-subject factor, respectively. The underlying idea was that if the difficulty in discriminating highly resembling faces was the main factor accounting for the absence of the self-face advantage in twins, the two groups should perform equally poorly with those stimuli. Results showed instead a main effect of group with Twins performing better than controls ((0.050 ± 0.003) vs. (0.037 ± 0.005); F_1,1_ = 5.351, p = 0.028, η^2^ = 0.160), an expected main effect of Orientation (F_1,1 =_ 113.588, p = 0.000, η^2^ = 0.802) and a significant interaction between the two factors (F_1,28 =_ 24.224, p = 0.000, η^2^ = 0.464). Newman-Keuls post-hoc comparisons showed the Twins were better in recognizing the self and co-twin faces with respect to control participants when stimuli were upright ((0.061 ± 0.013) vs. (0.041 ± 0.020); p = 0.002), but performed similarly when stimuli were inverted ((0.040 ± 0,012) vs. (0.033 ± 0.018); p = 0.294)) (Fig. 2B).

### The absence of self-advantage depends on how much twins report to physically resemble each other

**Fig 3:**
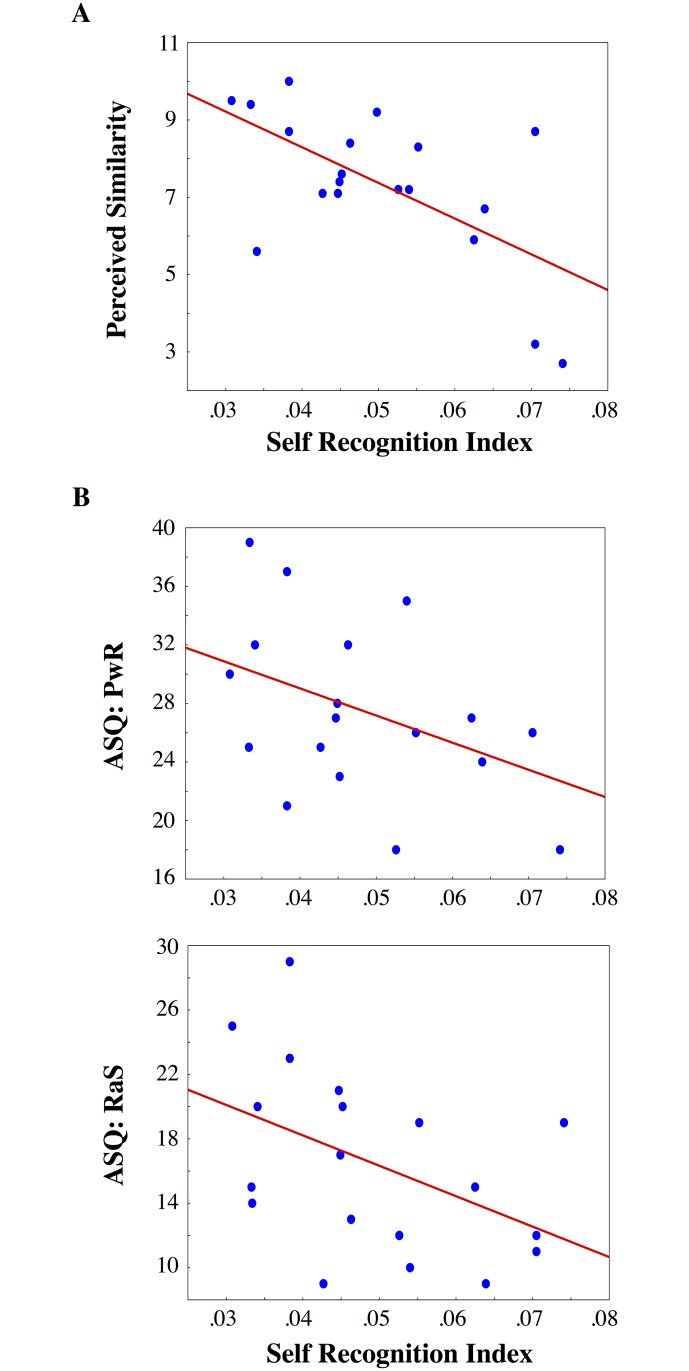
Correlations. A) Perceived Similarity and B) Insecure attachment (Preoccupation with Relationship (PwR); “Relationship as Secondary” (RaS) subscales of the Attachment Scale Questionnaire) were found to significantly predict the absence of self-face recognition advantage (normalized inverse efficiency scores mediated for upright and inverted Self-face, x-axes) in twins.

A standard regression model with “Interpersonal Perception” (‘Acquaintance’, ‘Frequentation’ and ‘Physical Resemblance’) as predictors were run on a self-recognition index (Self upright and upside-down normalized IE scores were mediated since results of the ANOVA showed that the absence of self-advantage was independent of stimuli orientation). After one outlier was removed (according to the Cook’s distance method [60]), the model showed to be significant (R^2^ = 0.49; F_3,15_ = 4.82; p = 0.015). Resemblance was the only significant variable. Indeed, the more the twins felt as if they physically resemble each other, the lower their performance at identifying their own face (B = -0.49; t(15) = -2.51; p = 0.024) (Fig. 3A). In other words, sharing highly similar physical features impaired twins’ ability to discriminate the self from the co-twin’s face.

To investigate the possible contribution of physical resemblance with other person’s face in the recognition task, we also checked if the controls' performance in self-face conditions (self upright and upside-down normalized IE scores were mediated, for the same reasons as above) was inversely related to how much they felt to physically resemble the twins, or if it depended on the level of acquaintance and frequentation with them. The model, however, was not significant (R^2^ = 0.47; F_3,15_ = 1.05; p = 0.467).

### The absence of self-advantage depends on twins’ anxious and avoidant attachment style

A standard regression model including the 5 subscales of the ASQ questionnaire (“Confidence”, “Discomfort with Closeness”, “Need for Approval”, “Preoccupation with Relationships” and “Relationships as Secondary”) as predictors were run on the self-recognition index (self upright and upside-down normalized IE scores were mediated since results of the ANOVA showed that the absence of self-advantage was independent of stimuli orientation). After one outlier removal according to the Cook’s distance method [60], the model proved to be significant (R^2^ = 0.56; F_5,13_ = 3.68; p = 0.027). There were two significant predictors: “Preoccupation with Relationships” and “Relationships as Secondary”. Thus the more the twins had an anxious approach to relationships (e.g. scored high on the “Preoccupation with Relationship”, B = -0.64; t(13) = -3.09; p = 0.009) and the more they had an avoidant approach to relationships, (i.e. they scored high on the “Relationship as Secondary” subscale, B = -0.72; t(13) = -3.15; p = 0.008) the lower their performance at identifying their own face (Fig. 3B).

The same model was tested on the control’s self-face performance but no significant results were found (R2 = 0.38; F5,4 = 0.50; p = 0.767).

## Discussion

In the present study we explored for the first time the ability of adult identical twins to recognize their own faces with respect to their co-twin’s face and the face of highly familiar individuals. This ability was also examined in control subjects who were highly familiar with the twins.

The results showed that the control group was better at recognizing their own face with respect to the twins’ faces, which were not discriminated between each other. This result is in line with previous literature indicating that there is something uniquely associated with self-recognition, and that, for control subjects, one’s own face has a particular status compared to other facial identities, regardless of their familiarity. Indeed, the self-face is recognized faster than both familiar and unknown faces [11,12,14–16,61].

Our study demonstrates that monozygotic (identical) twins do not demonstrate this self-face advantage, as their performance was comparable for self-face recognition and for the recognition of co-twin’s face. Tellingly, this (in)ability was predicted by the perceived physical similarity with the co-twin’s face and also by the tendency to have an insecure attachment style.

### Self-face recognition in twins

As previously mentioned, self-face has special status for the individual. In general, people have had daily exposure with their own faces for throughout most of their lives and are thus extremely familiar with seeing it. Moreover the self-face has a special salience and more emotional relevance than other faces. It is thus no surprise that personal familiarity, kinship, physical similarity, exposure, as well as the emotional salience of one's own face, influence the facial recognition processes [43–46] and impact the core network of visual areas as well as in extended cognitive and emotional systems [53,54]. Importantly, comparing the self-face with an identical co-twin’s face ensures that all the aforementioned factors are controlled for, and presents an opportunity for researchers to test the unique processes underlying visual self-face recognition. One recent study addressed this issue by investigating the electrophysiological correlates of the self- and dizygotic (non-identical) co-twin’s face [62]. This study showed that self and co-twin’s faces share very similar featural, configural, and matching processes, but differ with respect to the high-order stages of face processing. More specifically when the self-face was compared with a face associated with similar levels of lifetime exposure (and thus highly familiar and emotionally salient), the neural processes of visual self-recognition were similar at early stages indexed by analysis of visual components in 100–300 ms time range, which is sensitive to familiarity and learning effects. In contrast, unique processes seem to characterize later stages (in the 400–700 ms time-range) where the effects of facial-identity has important modulatory effects [63–66]. Also, the fact that self and twin recognition shared very similar electrophysiological signatures supports the ‘self-referent phenotype’ matching theory, i.e. that we recognize our kin by implicitly or explicitly comparing the similarity of other people’s appearance to our own [67]. Moreover, this may suggest that the degree of similarity in the neural processes for recognizing self and kin should also increase when the similarity between self and kin increases [53]. However, the ultimate confirmation of this prediction would come from the comparison of participants who view the self-face and the identical (i.e., monozygotic) twin’s face. Here, we show that, at least at a behavioral level (and with fast stimuli presentation), monozygotic twins fail to discriminate the self-face from the co-twin’s face and that the lack of self-face advantage is predicted by the reported levels physical similarity between the participant and the model. These results are congruent with two studies [17,65] that investigated the effect of physical similarity on self- and familiar- face recognition and found a specific impairment in self-face recognition when the self- and other's face were highly similar. Kircher et al. [22] compared the response times for recognizing the self and highly familiar, overlearned faces obtained by morphing a person’s own face or a friend’s face with a non familiar face. Results showed a significantly slower processing speed when the morphed face contained about 70–80% of the self (and 20–30% of the other face), but not when the self-face was morphed less extensively (with less than 15% of the other face) and thus was less ambiguous as compared to the overlearned face. Yoon and Kircher [68] compared recognition of self, familiar and unknown faces morphed with unknown faces, which were similar or dissimilar to the three identities. Results showed that there were delayed reaction times for the morphed images of "self versus similarly rated unknown faces" compared to the images of "self versus dissimilarly rated unknown faces". Such significant differences were not found for the other morphed versions.

Plausibly, this delayed recognition effect is due to a mismatch between the internal representation of the self-face and the observed self-similar face, artificially obtained with the morphing procedure. Such mismatch becomes relevant only in the case where self-similarity is involved. Indeed, the effect is selective for the self but not for the other's face, even when it is familiar or overlearned, suggesting the identity proof for the self is more complex and requires longer response times. In other words, we posit that, although processing the self has a special status, when the self-face is compared to another self-similar face the performance may be sub-optimal. This may explain why in the case when facial physical similarity becomes maximal (i.e. for monozygotic twins), participants are less able to discriminate the self- from the highly self-similar co-twin’s face to the point that their performance is lower even with respect to discriminating the face of a friend. However, given their high similarity to each other and dissimilarity from others, twins may still maintain a special representation of the self and of the co-twin face as a pair that take to a sort of “self-twin” processing advantage. We tested this hypothesis by comparing the performance of twins and friends (controls) when processing the co-twins’ faces. Twins were better than controls at processing their own and their co-twin upright faces (thus confirming our prediction). This comparison also ruled out the idea that a general difficulty in discriminating two faces with highly similar facial features was the only factor that could explain the lack of distinction between the two faces in both groups.

All in all, based on studies about the neural processing of the self- and dizygotic co-twin face [62], our pattern of results suggest that identical twins may rely upon similar neural processes for visual recognition of themselves and their co-twin, while non-identical twin participants may have to use different resources. Visual self-face recognition entails matching an image of the self to an internal representation of the self. Developmental and cognitive neuroscience studies have recently suggested [69,70] that internal representations of the self-face are built upon accumulating congruent multisensory experiences in which one’s own sensorimotor experience is matched with the face he/she sees in the mirror. This process ultimately allows one to self-identify with the face seen in the mirror [69–71]. The fact that the representation of one’s own face is based on the view that we perceive it in the mirror in everyday life has also been suggested by studies that found faster reaction times for the frontal rather than profile view of the self-face (while no such effect was found for familiar faces, which are usually perceived from many viewpoints) [72]. Also, the continuous updating of matching multisensory stimuli allows one to update the internal self-face representation and thus to maintain the sense of self-identity across gradual changes of one’s own facial appearance due to the passage of time. In monozygotic twins, the stored visual representations of the self and the monozygotic co-twin face, most likely overlap to a very high extent. Thus, we might speculate that self-recognition in twins might rely more heavily on the congruency of multisensory signals than on a stored visual representation of the self-face, which shares too many features with the co-twin’s face.

### Personality traits and face recognition in twins

Another novel result of the present study is that the lack of self-face advantage in identical twins was predicted by personality factors. The relationship between personality traits and face recognition in non-twin groups is not completely new. For instance, Platek and Gallup [47] studied a group of healthy volunteers and showed that left-hand reaction times to self-face images were positively correlated to scores of Schizotypal Personality Questionnaire, and that self-face processing in the right hemisphere was impaired in individuals with schizotypal traits. Again, Saito and colleagues showed how the “Big Five” factors “Extroversion”, “Neuroticism” and “Conscientiousness” are related to reaction times in a human face recognition task [73], a result partly replicated by Li and colleagues, who found that extroverts with better social skills are better able to recognize faces than introverts [48].

The current work extends these previous findings by showing a relationship between face recognition and attachment style in twins. While studies have investigated the relationship between attachment style and facial expression recognition [74–76], no data are available on the influence of attachment style over face identity recognition. The term ‘attachment style’ is influenced by early attachment experiences and refers to individual differences in emotion regulation and perceptions and beliefs about the self and significant others. Moreover, attachment style is broadly divided into two categories: secure and insecure. Here, we show how insecure attachment influences self-face recognition in couples of monozygotic twins: the recognition of self-faces within the Twin group was negatively predicted by higher “Preoccupation with Relationship” (PwR) and “Relationship as Secondary” (RaS) scores.

The PwR score is a core feature of anxious/ambivalent attachment style [77]. Individuals with high attachment-related anxiety display anxious and dependent approach to relationships, the tendency to obsess over maintaining their relationships with others [57], to feel unappreciated, and to be physically clingy toward their attachment figures [78,79]. Also, these individuals worry about their relationships because they fear abandonment and that they will be unable to cope alone.

Adults with high attachment-related avoidance (as indexed by high RaS scores) instead find it difficult to trust or depend on others, feel uneasy with emotional closeness and intimacy, are reluctant to ask their partner for support, believe that personal success is more important than relationships, and they give little importance on getting along with others [80].

It has been suggested that the “Preoccupation with Relationship” dimension incorporates a strong aspect of negative self-representation [57], while the dimension “Relationships as Secondary” incorporates aspects of a negative view of others [80].

Thus, we speculate that the tendency to confound the self with the co-twin face, as revealed by the face recognition task, could be related to different processes depending on having an insecure attachment, either anxious or avoidant. Probably, twins who reported higher presence of PwR could be more prone to mistaking themselves for their co-twin. In other words, at fast stimuli presentations, the confusion between the self and the co-twin face in the more “clingy” individuals could derive from a tendency to see their beloved co-twins instead of themselves. Recently, Davis and co-workers found that face recognition is negatively correlated with increasing social anxiety levels, indicating that higher social phobia is associated with lower face recognition [49]. In our study we found that another kind of social-related anxiety, i.e., PwR (see [81]), seems to play a role in influencing the recognition of very familiar and emotionally-significant faces (twins). Also, twins with highly anxious attachment may have negative self-representations, and it has been demonstrated that experimentally induced reduction of positive self-view through self-concept threat paradigm (i.e., by referencing negative traits to the self) may cancel out the self-face recognition advantage [82].

Our data also show that people with higher levels of RaS generally may not be so attentive to another person and they do not develop a special ability to identify the subtle differences that distinguish their own face from that of their co-twin. Thus we speculate that the self-other (i.e., co-twin) confusion we found in the present experimental task may arise from the twins' tendency to mistake the co-twin for themself.

## Conclusions

Overall our results reveal that, at least for the rapid stimuli presentation used in our study, monozygotic twins do not show the visual self-face recognition advantage. It is important to note that twins lack a self-face advantage with respect to the co-twin’s face and that it is highly plausible to find facilitated self-face recognition if the self-face is compared to other non-twin faces.

We hypothesize that such lack of self-advantage may depend on overlapping visual representation of the self- and the highly self-similar co-twin face. Indeed, the effect varies as a function of perceived physical resemblance. Furthermore, the inability to discriminate the self from the co-twin is related to insecure attachment styles, which defines perceptions and the beliefs about self and significant others.

We have to acknowledge that the current study was also not free of limitations: given the high specificity of the study population, the sample size was limited. Also, although we did our best to match twins and controls in sex, age and every day frequentation, some of the controls did not know the twins from birth. Therefore, future studies may take into account these factors to further control the possible role they play in face recognition in identical twins.

## Supporting Information

S1 Dataset (XLS)
